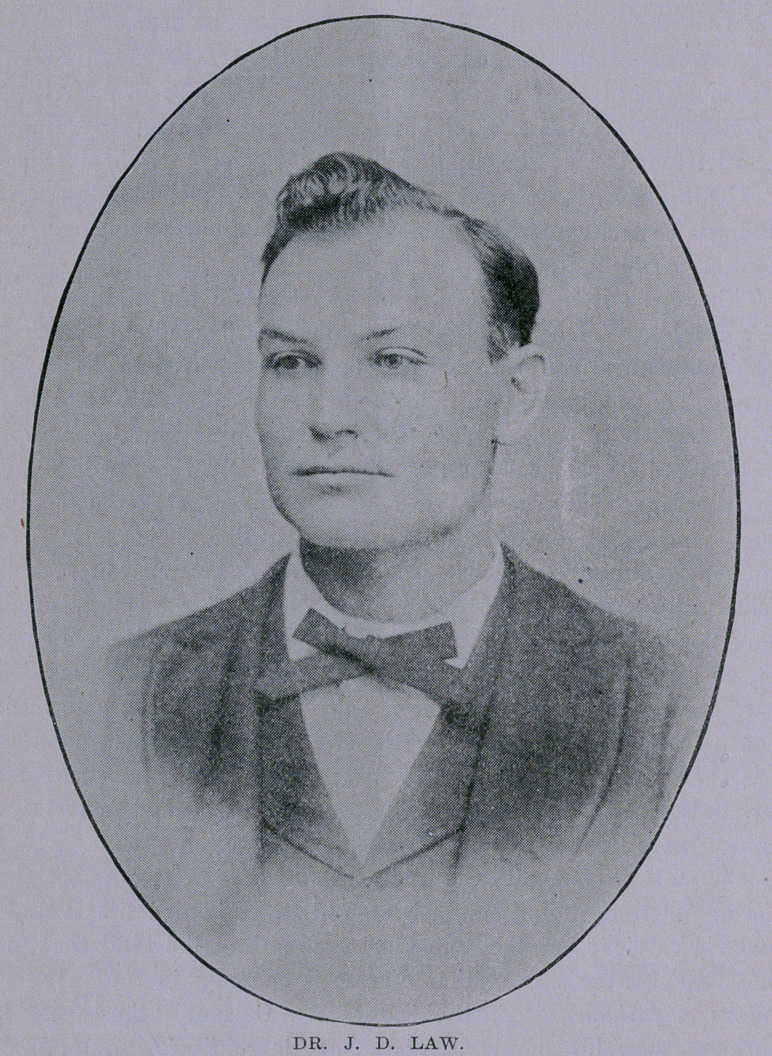# Death of Dr. Law

**Published:** 1908-02

**Authors:** 


DEATH OF DR. LAW.—It is with pain and profound regret that we announce the death of one of Texas' ablest, most distinguished and popular physician, Dr. Jarrette D. Law, of Belton. He died at his home Tuesday morning, January 21st (ult.), after a brief illness, at the early age of 47 years.

Dr. Law was one of the most scholarly and accomplished physicians of the State. He possessed all the qualities that go to make up the true physician, and he was held in the highest respect by all classes. His funeral was an outpouring of the population of Bell county, irrespective of class or conditions, the largest ever witnessed in Bell or any other county. Business was suspended, and the city was in mourning. Those of our guild who heard his polished, eloquent and scholarly address at the Houston meeting of the Texas State Medical Association in April, 1905—the "Annual Oration," which he was invited to deliver—will never forget either the subject, "The Physician and the Humanities," or the splendid orator. Peace to his ashes. He will live in the hearts and memories of all who had the honor of his friendship and the good fortune to know him as citizen, physician, man, father and Christian gentleman. Dr. Law was a self-made man. He was a native of Louisiana, and was born in Red River Parish, that State, January 29, 1861. His literary education was acquired at Salado College, Bell county, and his medical at Louisville Medical College, class of 1884, where he graduated with first honors. In 1886 he married Mildred Barton, of Salado, and she and five children survive him.

## Figures and Tables

**Figure f1:**